# Sanity of Mind

**Published:** 1901-05

**Authors:** 


Sanity of Mind. A Study of its Conditions, and of the Means to its Development and Preservation. By David F. Lincoln, M. D., Author of "School and Industrial Hygiene," etc. Duodecimo, pp. 183. New York and London: G. P. Putnam's Sons. 1900. [Price, $1.25]

This little book is a vigorous, healthy protest against fatalism. The thesis that characters are developed by self is maintained by the author. He has no doubt that the individual has a share in character building and believes that he has the power of choosing between different ways of spending his time.

As he says: "If we are living under the fatalistic impression which is so easily brought on by hearing of one or two good cases of heredity, the whole argument of self-control will take a goody-goody aspect, and will seem a perfunctory preachment on the author's part."

The chief practical value of this book is in chapter four. Here he discusses education based upon the study of development. He gives attention to bodily training; the teaching of the feeble-minded and the backward; and the effects of over study. On this last point the author states that in the United States and Canada the question of overwork, as regards the amount of time involved, is practically settled among intelligent educators. He contends that the food question is of more importance, for it is now the fashion to concentrate all the work between 8.30 and 1.30. The result has been, as one prominent educator reported, "that the last hour of the morning is practically time thrown away—it is merely the spurring on of the exhausted." This can be obviated, as at the Pratt Institute, by serving a proper hot lunch in the middle of the forenoon.

The object of the book is the discussion of what tends to promote sanity of mind and what tends to destroy it. His definition is new and strong; "sanity is harmony with one's psychic environment." The author wisely contends that the lesson of self-control must be learned by the young, for the habit self-control is of the greatest importance as a means of checking tendencies to mental disorder. Of disobedience he says "it is a mental habit which predisposes to mental derangement."

It is a temptation to quote freely from the author's terse sentences, so full is the book of wise counsel, and scientific psychology. We earnestly recommend it to teachers, parents and physicians.

